# Copy number of latent viruses, oncogenicity, and the microcompetition model

**DOI:** 10.18632/oncotarget.25804

**Published:** 2018-08-03

**Authors:** Hanan Polansky, Hava Schwab

**Affiliations:** The Center for the Biology of Chronic Disease, Valley Cottage, NY, USA

**Keywords:** cancer and EBV, microcompetition, transcription factor, gene expression, viral load

A series of experiments described in a recent study showed that the copy number of EBV in a cell during latency is strongly associated with oncogenicity [1]. Specifically, this number refers to the genome copy number in a single cell and not the total copy number of the virus in the infected host. According to the authors: “The copy number in a single cell or average level seems more paramount than general level.” Based on their results the authors conclude: “In this study, we present the first evidence by experimental model that the copy number of EBV is an important factor involved in the viral pathogenesis. We thus speculate that one cell with very low EBV copy number might not be transformed easily and develop a cancer. This may partly explain the fact that the ubiquitous virus is associated with only a minority of cancer.”

The results of these experiments, and the conclusions by Zuo et al., are consistent with the Microcompetition Model first described in the book entitled ‘Microcompetition with Foreign DNA and the Origin of Chronic Disease’ [2, 3].

The model concentrates on certain viruses, such as the Herpes Simplex Virus 1 (HSV-1), Epstein- Barr virus (EBV), Cytomegalovirus (CMV), Human T-cell lymphotropic virus (HTLV), and Human Immunodeficiency Virus (HIV), that include a strong cis-regulatory element in their promoters/enhancers, called the N-box. This cis-element binds the cellular GABP·p300 transcription complex during the latent phase. Since the level of the GABP·p300 is limiting, the viral N-boxes reduce the availability of the complex for to the cellular genes. The lower pool of GABP·p300 causes the cellular genes to express abnormal levels of their proteins. The genes that are transactivated by the GABP·p300 complex synthesize fewer proteins. The genes that are suppressed by the complex synthesize more proteins. The abnormal levels of these cellular proteins cause many common chronic diseases, including cancer and diabetes.

For example, the breast cancer associated gene type 1 (BRCA1) promoter has three N-boxes in its -200 to -178 region. GABP has been shown to interact with the N-boxes and stimulate transcription of the gene [4].Transcriptional inactivation of BRCA1 increases cell proliferation and leads to breast cancer [5]. The latent virus promoters sequester the limited amount of GABP·p300 available to the BRCA1 promoter, leading to decreased transcription of the gene, ultimately leading to breast cancer. This shows how a virus that includes a binding site for GABP·p300 can cause cancer.

It is well known that there is a balance between the efficiency of the immune system and the copy number of latent viruses in the host. It is also known that many events can decrease the efficiency of the immune system, including, aging, certain medications, surgery, chemotherapy, radiation, and stress [6]. When such an event occurs, the efficiency of the immune system decreases, the copy number of the latent viruses increases, and the risk of cancer also increases, as observed by Zuo et al.

To summarize, Zuo et al. conclude: “It has been noticed that EBV load in tumor tissues or blood is associated with the clinical progression and prognosis in both lymphoma and NPC. Our result verifies this association. We also emphasize the importance to measure the level of gene expression or copy number in the virus study instead of only concerning ‘with and without’.” In this paper we show that our interpretation of the Microcompetition Model supports this conclusion. We also believe that the copy number of the latent viruses that include an N-box in their promoter/enhancer, including EBV, and not the ‘infected or not infected’ status is an important factor that determines the pathogenesis of cancer, and therefore should be tested in clinical practice.
